# Herpes Simplex Esophagitis in an Immunocompetent Adult

**DOI:** 10.7759/cureus.77293

**Published:** 2025-01-11

**Authors:** Richard M Bresler, Zachary Ellis, Foster West, Kellie M Mitchell, Jacob Whelan

**Affiliations:** 1 Internal Medicine, Baptist Memorial Hospital-North Mississippi, Oxford, USA

**Keywords:** acyclovir, esophagitis, herpes simplex virus, immunocompetent, odynophagia

## Abstract

Herpes simplex esophagitis (HSE) caused by herpes simplex virus (HSV) infection is a well-recognized opportunistic infection in an immunocompromised host. HSE in immunocompetent patients is an uncommon clinical event; however, when it does occur, it is generally a self-limiting condition. This case involves an immunocompetent 19-year-old female patient who presented to the emergency department (ED) with a three-day history of nausea, vomiting, and inability to tolerate oral intake. The diagnosis of HSE was made via histopathological analysis and HSV-1 polymerase chain reaction (PCR) of an esophageal biopsy. The patient clinically improved on acyclovir. HSE in the immunocompetent patient is a rare clinical entity. A combination of history, upper endoscopy, histopathology, and HSV-PCR is used to diagnose HSE. Antiviral therapy with acyclovir may reduce the severity and duration of infection.

## Introduction

Herpes simplex esophagitis (HSE) is a clinical entity commonly seen in immunocompromised patients [1]. It is often found in patients with acquired immunodeficiency syndrome (AIDS) or malignancies or those receiving immunosuppressive therapy [1-3]. In contrast, HSE in immunocompetent patients is rarely reported in the literature and may represent primary disease or the reactivation of a latent infection [4]. Clinically, it usually presents as symptoms of acute odynophagia, fever, and retrosternal chest pain. The diagnosis requires a high index of suspicion in the immunocompetent patient. This case involves a 19-year-old female patient with no significant past medical history who presented to the emergency department (ED) with a three-day history of nausea, vomiting, and inability to tolerate oral intake. HSE was diagnosed endoscopically with the histopathological examination of an esophageal biopsy. The patient demonstrated significant clinical improvement on acyclovir.

## Case presentation

A previously healthy 19-year-old Caucasian female patient presented to the ED with a three-day history of nausea, vomiting, and inability to tolerate oral intake due to dysphagia. She denied fevers, chills, weight loss, lymphadenopathy, chest pain, odynophagia, dyspnea, cough, or abdominal pain. Medical, social, and family history were unremarkable. She reported infrequent alcohol consumption and denied any illicit substance or tobacco use. She had no recent sick contacts; she did have a cat in her home. She was sexually active, in a monogamous relationship, and current on all routine childhood vaccines and the human papillomavirus (HPV) vaccine.

**Figure 1:**
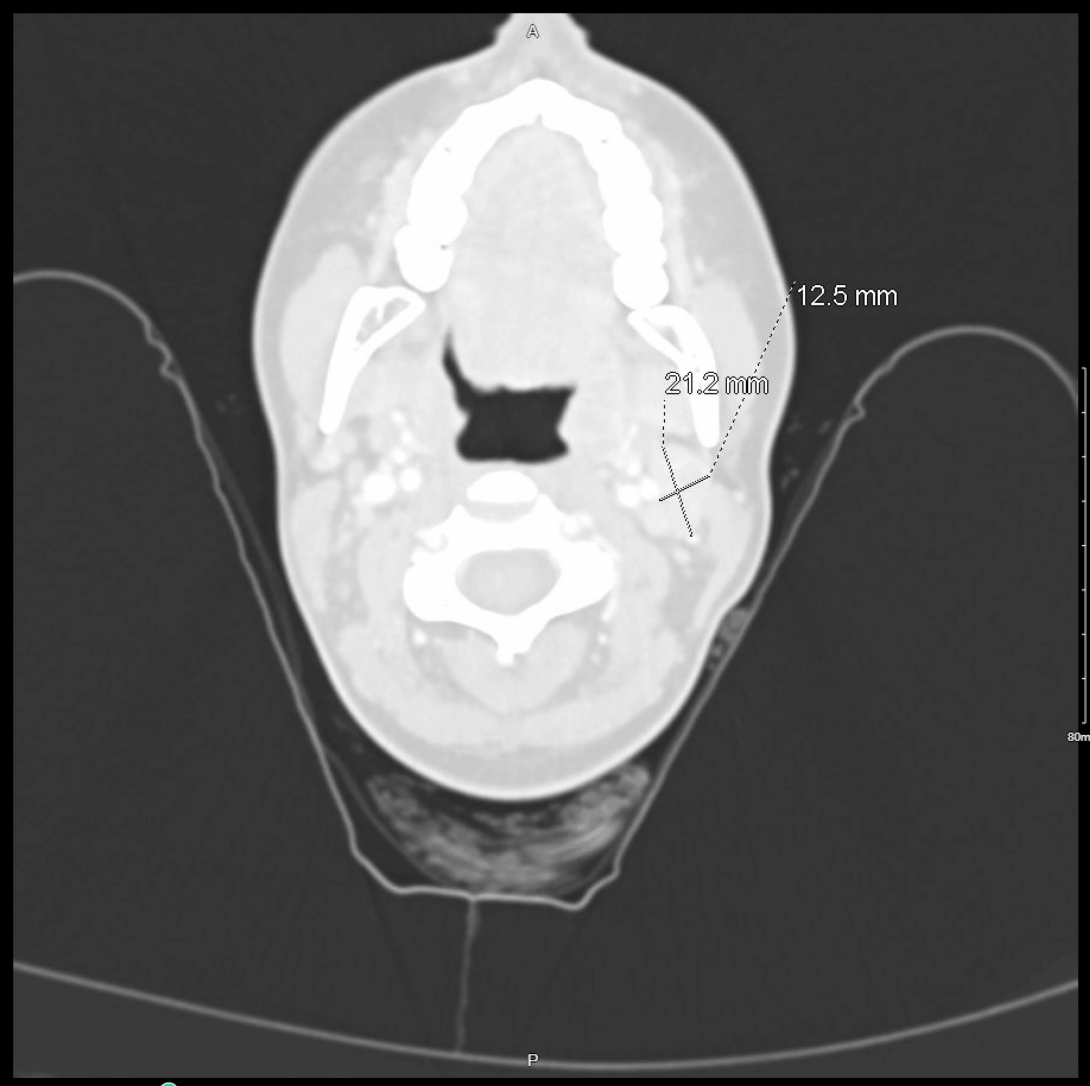
CT of the soft tissue of the head and neck with contrast revealing a borderline enlarged left cervical lymph node measuring 2.1×1.2 cm CT: computed tomography

Vital signs on admission were the following: temperature of 36.3°C, heart rate of 102, respiratory rate of 22, and oxygen saturation of 99% on room air. Physical exam findings were unremarkable; there was no palpable head or neck lymphadenopathy on the admission exam. Labs on admission were notable for monocytosis (Table 1). The ED ordered computed tomography (CT) of the head and neck to evaluate for possible etiologies of dysphagia; CT revealed a borderline enlarged left cervical lymph node posterior to the left angle of the mandible measuring 2.1×1.2 cm (Figure 1). Initial treatment consisted of intravenous ceftriaxone 1 g every 24 hours and intravenous fluids due to concern for acute bacterial pharyngitis. The patient started to develop low-grade fevers one day after admission. The Infectious Disease (ID) service was consulted. Serological studies for Epstein-Barr virus (EBV), human immunodeficiency virus (HIV), hepatitis B, and hepatitis C were ordered by ID, all of which were negative. Blood cultures returned negative. There was suspicion of cat scratch disease, given the enlarged cervical lymph node on the CT scan and history of exposure to cats. Therefore, intravenous azithromycin 500 mg every 24 hours was added to intravenous ceftriaxone; cat scratch disease was later ruled out with negative serologies for *Bartonella*. Despite not having a definitive diagnosis, the patient had made significant clinical improvement with reported near resolution of her symptoms and was therefore discharged four days post-admission with an empiric course of azithromycin 250 mg daily for four days and Augmentin 875-125 mg twice daily for 10 days. 

**Table 1:** Significant labs during the first admission

Lab	First admission	Reference range
White blood cells	9.3	5-10 K/uL
Hemoglobin	15.1	12-16 g/dL
Platelets	258	150-500 K/uL
Monocytes	6.6%	0-6%

**Figure 2:**
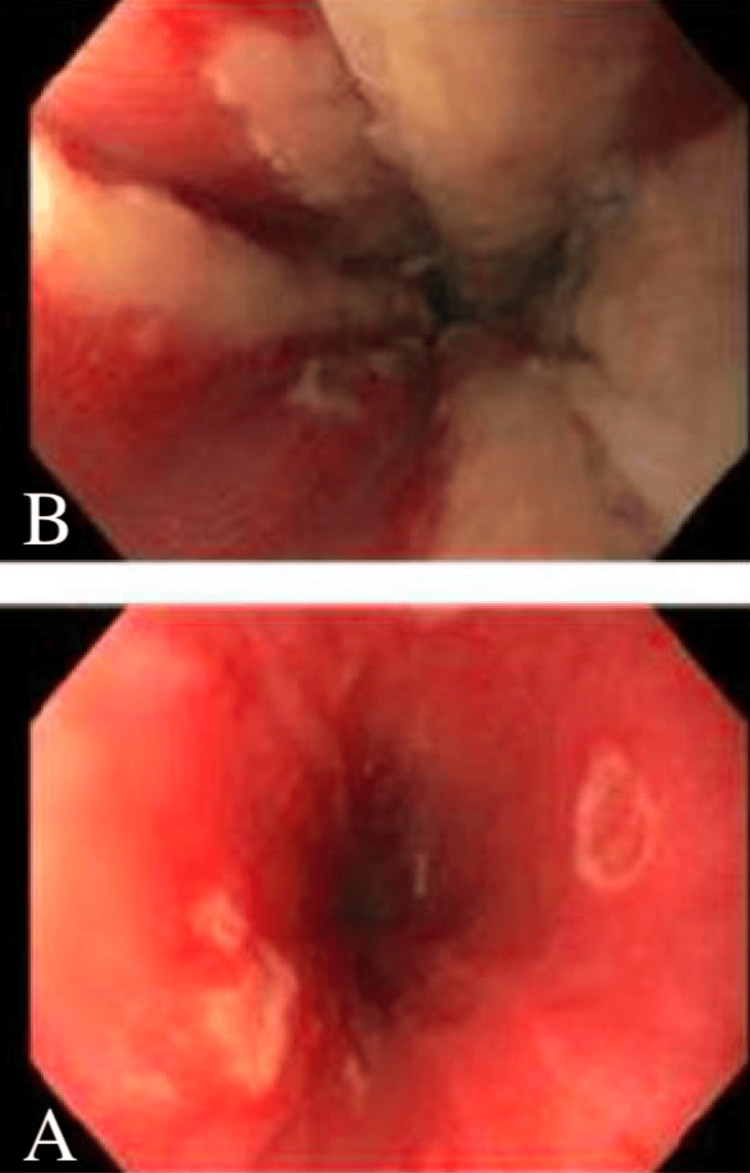
(A) Multiple discrete shallow ulcers in the middle third of the esophagus. (B) Friable mucosa, erythema, and exudate were present in the lower third of the esophagus

**Figure 3:**
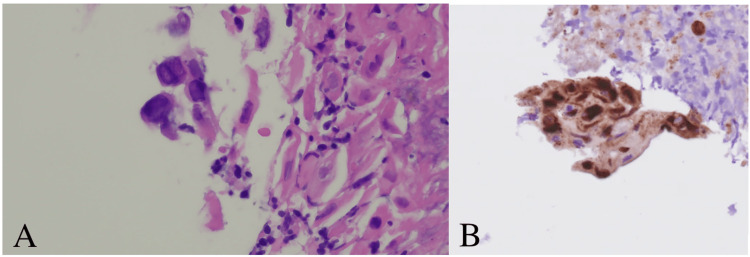
(A) Histological features of HSE showing Cowdry A intranuclear inclusion bodies in infected epithelial cells (H&E, ×400). (B) Immunohistochemistry technique with positive immunostaining demonstrating intranuclear inclusions (anti-HSV-1 antibody, ×200) HSE: herpes simplex esophagitis; H&E: hematoxylin and eosin; HSV: herpes simplex virus

One day after hospital discharge, she presented to the ED with recurrent nausea, vomiting, and inability to tolerate oral intake due to worsening dysphagia. Vital signs on admission were the following: temperature of 37.1°C, heart rate of 91, respiratory rate of 19, and oxygen saturation of 98% on room air. Physical exam findings were unremarkable. Labs on admission revealed a worsening monocytosis (Table 2). The patient was restarted on intravenous azithromycin 500 mg every 24 hours and ceftriaxone 1 g every 24 hours, given her initial improvement on these agents prior to discharge. Given her persistent symptoms of dysphagia and newly reported odynophagia upon further questioning, she underwent esophagogastroduodenoscopy (EGD) one day after readmission. The EGD revealed discrete shallow ulcers in the middle third of the esophagus (Figure 2A). Friable mucosa, erythema, and exudate were present in the lower third of the esophagus (Figure 2B). Histology showed eosinophilic round intranuclear bodies (Cowdry A inclusion bodies) (Figure 3A) with positive herpes simplex virus (HSV)-1 immunostaining (Figure 3B). HSV-1 PCR of an esophageal biopsy was positive. Further serological studies revealed a positive HSV-1 polymerase chain reaction (PCR) and HSV IgM antibody. HSV IgG and cytomegalovirus (CMV) IgG were negative. The diagnosis of HSE was made, and intravenous antibiotics were discontinued. The patient was started on intravenous acyclovir 5 mg/kg every eight hours and experienced mitigation of her nausea and vomiting. She continued treatment with oral acyclovir 400 mg three times daily for a total of 14 days when she could tolerate oral intake. She obtained resolution of her symptoms with appropriate treatment of HSE. 

**Table 2:** Significant labs on readmission

Lab	Readmission	Reference range
White blood cells	8.3	5-10 K/uL
Hemoglobin	15	12-16 g/dL
Platelets	210	150-500 K/uL
Monocytes	9.3%	0-6%

## Discussion

HSV is a DNA virus known to cause visceral disease in immunocompromised patients. The most common visceral organ affected by HSV is the esophagus [5]. HSE in the immunocompetent host is a rare clinical entity. It is more often due to a primary infection rather than the reactivation of a latent infection [6]. The pathogenesis is thought to be secondary to the local spread of the virus from an oropharyngeal infection into the esophagus or the spread of the virus to the esophageal mucosa via the vagus nerve [7]. The diagnosis requires a high degree of suspicion in the immunocompetent patient. The typical patient presentation is a young male with symptoms of acute odynophagia, fever, and retrosternal chest pain. Upper respiratory symptoms and orolabial herpetic lesions may also be present [8]. In this case, the patient did not present on admission with the aforementioned symptoms and was initially thought to have cat scratch disease; only upon EGD finding was the diagnosis of HSE suspected.

The lower or mid esophagus is more likely involved than the proximal esophagus in HSE [8]. The characteristic findings are multiple small ulcers, friable mucosa, and erythema that ascends from the lower esophagus [8]. Exudate is present in the majority of cases [8]. An upper endoscopy with tissue biopsy samples for histological examination and viral culture or PCR techniques are required for diagnosis. Biopsies from the edge of the ulcers provide the best diagnostic yield for histopathological examination and viral culture [9]. In our patient, the diagnosis was confirmed with hematoxylin and eosin (H&E) staining and immunohistochemistry. Typically, histopathology shows multinucleated giant cells with or without eosinophilic intranuclear inclusions (Cowdry inclusion bodies) [9]. Also, HSV-1 DNA was detected through PCR of an esophageal biopsy. Serology usually shows an acute infection pattern with positive IgM antibody and negative IgG antibody [8]. Serology is of limited value as a majority of healthy individuals will have had prior exposure to HSV. However, seroconversion may be diagnostic [8]. Given that HSE is more frequent in immunosuppressed patients than in immunocompetent patients, ruling out an underlying immune disorder such as HIV infection is imperative. In the majority of cases, HSE usually spontaneously resolves in immunocompetent hosts after one to two weeks even without treatment [10]. However, gastrointestinal bleeding or esophageal perforation secondary to HSE has been rarely reported in the literature [11,12]. Acyclovir 400 mg five times a day for 14-21 days is the standard treatment for HSE in the immunocompetent host [10]. Given the rare incidence of HSE in the immunocompetent host, no clinical trial has been performed to examine the efficacy of acyclovir in HSE in immunocompetent patients. Despite the evidence, our patient significantly improved upon the initiation of acyclovir. The use of acyclovir in immunocompetent patients with HSE has shown promise in attenuating the severity of infection and hastening recovery [13].

This is a case of an immunocompetent 19-year-old Caucasian female patient who presented for the evaluation of intractable nausea and vomiting with decreased oral intake secondary to significant dysphagia. During index hospitalization, she had initial improvement in symptoms with supportive care; however, shortly after discharge, she had a recurrence and acute worsening of her presenting symptoms. Gastroenterology consultation and EGD ultimately revealed her diagnosis. Upon histological and serological confirmation of HSE, despite the initial history being unrevealing, a detailed review of the patient's sexual history revealed recent oral sex with a new partner. Her symptoms ultimately resolved with supportive care and a course of acyclovir. 

## Conclusions

HSE in the immunocompetent patient is a rare clinical entity. Ruling out an underlying immunosuppressive disorder is imperative. The diagnosis is dependent on a high index of suspicion. A combination of history, upper endoscopy, histopathology, and HSV-PCR is used to diagnose HSE. Serology for HSV should only be obtained in patients suspected of having primary HSV infection. Antiviral therapy with acyclovir may reduce the severity and duration of infection. This case reveals the importance of including HSE in the differential for an otherwise healthy patient with acute-onset dysphagia or odynophagia. 
